# Fast Extraction and Detection of 4-Methylimidazole in Soy Sauce Using Magnetic Molecularly Imprinted Polymer by HPLC

**DOI:** 10.3390/molecules22111885

**Published:** 2017-11-02

**Authors:** Zufei Feng, Yan Lu, Yingjuan Zhao, Helin Ye

**Affiliations:** 1 Department of Applied Chemistry, School of Science, Xi’an University of Technology, Xi’an 710061, China; zufeifeng@xaut.edu.cn (Z.F.); 18829039398@163.com (Y.L.); zhaoyj@xaut.edu.cn (Y.Z.); 2 School of Chemistry and Environmental Science, Lanzhou City University, Lanzhou 730070, China

**Keywords:** 4-methylimidazole, magnetic molecularly imprinted polymer, soy sauce samples, high performance liquid chromatography

## Abstract

On the basis of magnetic molecularly imprinted polymer (MMIP) solid-phase extraction coupled with high performance liquid chromatography, we established a new method for the determination of the 4-methylimidazole (4-MEI) in soy sauce. Scanning electron microscopy (SEM), Fourier transform infrared (FT-IR), X-ray diffraction (XRD) and vibrating sample magnetometer (VSM) were used to characterize the synthesized MMIPs. To evaluate the polymers, batch rebinding experiments were carried out. The binding strength and capacity were determined from the derived Freundlich isotherm (FI) equation. The selective recognition capability of MMIPs was investigated with a reference compound and a structurally similar compound. As a selective pre-concentration sorbents for 4-methylimidazole in soy sauce, the MMIPs showed a satisfied recoveries rate of spiked samples, ranged from 97% to 105%. As a result, the prepared MMIPs could be applied to selectively pre-concentrate and determine 4-methylimidazole in soy sauce samples.

## 1. Introduction

Soy sauce, which is made by soybean and wheat after the pretreatment of starter-making, fermentation, and heating preparation process, is one of the traditional condiments in China, as well as one of the most popular condiments worldwide. Indeed, it is the third popular condiment in the USA (after mayonnaise and ketchup), with a market share worth of 725 million dollars in 2014 [1]. Soy sauce contains plentiful amino acid, soluble protein, sugars, organic acids and other nutrients. But under the condition of heating, these reducing sugar, amino acid or protein may carry out Maillard Reaction and Caramelization Reaction. These chemical reactions can not only produce the flavor substances, but also the small molecules of heterocyclic compounds, such as 4-methylimidazole (4-MEI) [2].

Chinese national standard divided the soy sauce into two types: fermented soy sauce and preparation soy sauce. However, preparation soy sauce should contain the fermented soy sauce as the main part, more than 50% [3]. The color and luster are major sensory indexes of the soy sauce. The color and luster of fermented soy sauce are mainly produced in the fermentation process; meanwhile the color and luster of soy sauce partly come from the additive of caramel color. Caramel color is widely used in the soy sauce, vinegar, rice wine, beer, beverage and some of the baked food. When using the ammonium sulfite as raw material to produce caramel color, nitrogen heterocyclic compounds, such as 4-MEI will be produced as the by-product. 4-MEI is the important factor that affects the safety of caramel color. 4-MEI can cause high risk in the case of large doses.

As a neurotoxic agent [4], 4-MEI can inhibit the cytochrome P450 isoenzyme P450WE1 which catalyses the oxidation of many known or suspected carcinogens of low molecular mass in the human liver in some vitro studies [5]. In addition, 4-MEI can induce alveolar/bronchiolar adenoma and carcinoma in both male and female mice according to a toxicological study leaded by the National Institute of Environmental Health Sciences of USA [6]. Also, TOX-67 test shows that it can cause cancer [7]. According to the Food Chemicals Codex (FCC V-2004, USA), 4-MEI in caramel is limited to less than 0.025%. Chinese government standard (GB8817-2001) stipulates that 4-MEI should be no more than 0.02%. Consequently, it’s a matter of great urgency to establish a sensitive, simple and direct method to extract and detect the 4-MEI in soy sauces for safe use.

The methods to measure 4-MEI in the soy sauce include ultraviolet-vis spectrophotometers (UV) [8], gas chromatography (GC) [7], liquid chromatography (LC) [9,10], gas chromatography-mass spectrometry (GC-MS), gas chromatography-tandem mass spectrometry (GC-MS/MS) and liquid chromatography-tandem mass spectrometry (LC/MS/MS) [11,12,13,14]. Due to the small molecule and complex matrix in the real samples, the accuracy of UV is poor; most of GC methods need the derivatization [15]. Although chromatography-mass spectrometry also possesses the advantages of high separation and identification ability, the preparation of sample takes plenty of time; and the cost of all the experimentation is probably high. On another hand, the low penetration of LC-MS in most of Chinese laboratory constrains the practicability of this detection method. 

In the presence of a template molecule, molecularly imprinted polymers (MIPs) were synthesized with a functional monomer and a cross-linker by co-polymerization method. MIPs possess selective molecular recognition ability due to the tail-made recognition sites [16]. Firstly, the template molecule and the functional monomer are bonding together due to intermolecular force; then the functional monomers are polymerized in the presence of cross-linking agent and initiator; when the template molecule is removed, the binding site which is complementary with in size, geometry and functional groups is exposed in the polymer matrix [17,18]. As a result, the final network displays significantly selectivity and affinity for the template than for its structural analogues [19,20]. The magnetic polymers not only possess the advantage of easily collected and fast separated by an external magnetic field without additional centrifugation or filtration, but also promote the selective recognition due to the specific ability of MIPs.

This study first reported a method to recognize 4-MEI in soy sauce samples with a purposive synthesized magnetic MIPs. After highly enrichment, HPLC-PAD can provide satisfied sensitivity, recoveries, time efficiency and economical efficiency.

## 2. Materials and Methods

### 2.1. Materials and Reagents

Some chemical reagents were purchased from Reagent Company: 4-MEI from Alfa Aesar (Tianjin, China); Acrylamide (AM), styrene (St), ethylene glycol dimethacrylamide (EGDMA), and 2,2′-azobisissobutyronitrile (AIBN) from Alfa Aesar (Tianjin, China); both Ferric chloride (FeCl_3_·6H_2_O) and ferrous chloride (FeCl_2_·4H_2_O) from Fuchen Chemical Reagents Factory (Tianjin, China); Chromatographic grade methanol and acetonitrile from Merck Co. (Darmstadt, Germany); Dimethyl sulfoxide (DMSO), polyethylene glycol (PEG-6000), acetic acid, ammonium hydroxide and the other chemicals from Tianjin Chemical Reagent Co. (Tianjin, China). Ultrapure water was prepared by an ultra purification water system. Two soy sauce samples were obtained from a supermarket in Xi’an, named Shengchou (light color; Batch No: 4710; Zhengzhou Jiajia Flavor Industry Co., Ltd., Zhengzhou, China) and Laochou (dark color; Batch No: 20130820H; Jiangsu Hengshun Vinegar Industry Co., Ltd., Zhenjiang, China). All HPLC solutions were filtered through a 0.45 μm filter before use.

### 2.2. Instrumentation 

A WATERS Series (WATERS Technologies, Milford, MA, USA) LC system equipped with an e2695 Alliance Quaternary Pump, a 2998 Photodiode Array Detector (PAD), an Alliance Col Heater column oven and an automatic sampler were employed in this study. The system was controlled by an Empower 2 Personal Single System. A Sino-Chrom ODS-AP column (5 µm, 230 mm × 4.6 mm) (Dalian Elite Analytical Instruments Co., Ltd., Dalian, China) was used. For the analytical chromatography, isocratic elution with methanol and KH_2_PO_4_ (0.05 mol L^−1^) in a ratio of 12:88 (*v*/*v*) was performed. The flow rate was 1.0 mL min^−1^. The detection wavelength and column temperature were set at 233 nm and 28 °C, respectively. The loading volume was 20 µL. A model FE20 Plus pH meter (Mettler-Toledo, Shanghai, China) equipped with InLab^®^ Micro pH combination electrodes (Mettler-Toledo, Schwerzenbach, Switzerland) was used to measure the pH value. X-ray diffraction (XRD) were measured using a Bruker D8 Advance (XRD, Bruker, Germany). A Lake Shore 7307 vibrating sample magnetometer (VSM) (Lakeshore, Westerville, OH, USA) was used to measure the magnetic properties. SEM image was obtained via Hitachi S-4800 field emission scanning electron microscope (Tokyo, Japan). A Hitachi H-7650 transmission electron microscope (TEM, Hitachi, Tokyo, Japan) was used. A Nicolet Nexus-670 FT-IR spectrometer was used to obtain the FT-IR spectra, with the wave numbers ranged from 500 cm^−1^ to 4000 cm^−1^.

### 2.3. Preparation of Fe_3_O_4_ Magnetic Particles

Chemical co-precipitation method with slightly modification according to previously study [21] was used to synthesize the Fe_3_O_4_ magnetic particles. In a 250 mL three-necked flask, 15 mmol FeCl_3_·6H_2_O and 10 mmol FeCl_2_·4H_2_O were dissolved with 80 mL of deoxygenated water. Then the solution was constantly stirred. 

The ammonium hydroxide solution (50 mL, 5%) was added drop by drop when the temperature was raised to 60 °C. Then the mixture was stirred vigorously for 60 min at 300 rpm, 60 °C. The whole reaction process was carried out under nitrogen protection. After the reaction, washed the reactant several times with pure water and a magnet was used to collect the Fe_3_O_4_ MNPs.

Surface modifier was used to modify the surface of Fe_3_O_4_ particles. Fe_3_O_4_ (2.0 g) and PEG (10.0 g) were dissolved in deionized water (30 mL) by stirring for 20 min. Then sonicating for 30 min, the solution became homogeneously dispersed.

### 2.4. Preparation of the MMIPs 

According to the previous study [22], the MMIPs were synthesized with a slight modification. The template (4-MEI, 1 mmol) and the functional monomer (AM, 6 mmol) were dissolved in 50 mL of acetonitrile. After stored in dark for 18 h at room temperature, the pre-polymerization solution, PEG-Fe_3_O_4_ particles, dispersing media (doubly distilled water, 80 mL), copolymer monomer (St, 79.6 mmol), cross-linker (EGDMA, 30 mmol) and initiator (AIBN, 0.6 mmol) were well mixed in a 250 mL three-neck flask. The mixture was degassed in an ultrasonic bath for 15 min. Stirring at 300 rpm and 70 °C, the polymerization reaction has taken 22 h under nitrogen protection. After collected by an extra magnetic field, MMIPs were washed by a solution of methanol/acetic acid (9:1, *v*/*v*) to remove the templates, followed by methanol. Finally, the particles were dried in vacuum. The same method was used to prepare the MNIPs, but without the template.

### 2.5. Adsorption and Selectivity Evaluation

In order to evaluate the recognizing and binding capacity of MMIPs for in methanol, adsorption test was performed. A series of concentrations of 4-MEI solution (5–300 mg L^−1^, 1 mL) was prepared in 2 mL centrifuge tube. Then 20 mg MMIPs or MNIPs were suspended in these series of solution tubes, and shaken for 15 min at 25 °C. MMIPs or MNIPs was separated from the solution by a magnet deposited outside of the sample tube. Following, the supernatant was determined by HPLC. Deducted the amount of free 4-MEI from the amount added, the amount of 4-MEI binding to the MMIPs or MNIPs was obtained by calculation. In order to evaluate the binding parameters of the MMIPs and MNIPs, the data of the adsorption experiment were further processed refer to the Freundlich isotherm (FI) model.

Compared with reference compounds salicylic acid and benzoic acid, three different concentrations (20, 60, 120 µg L^−1^) were set to investigate the selectivity of the synthesized sorbent.

### 2.6. Extraction Procedure 

20 mg of MMIPs were added directly into 1 mL of soy sauce sample, and then shaken for 15 min. MMIPs were separated from the sample solution by a magnet. One milliliter of acetonitrile/formic acid (9:1, *v*/*v*) was used to elute the MMIPs by sonication for 15 min. Evaporated dryness from 0.5 mL of supernatants were dissolved into 0.1 mL of methanol, followed by HPLC-PAD analysis.

## 3. Results and Discussion

### 3.1. Preparation and Characterization of MMIPs 

**Figure 1:**
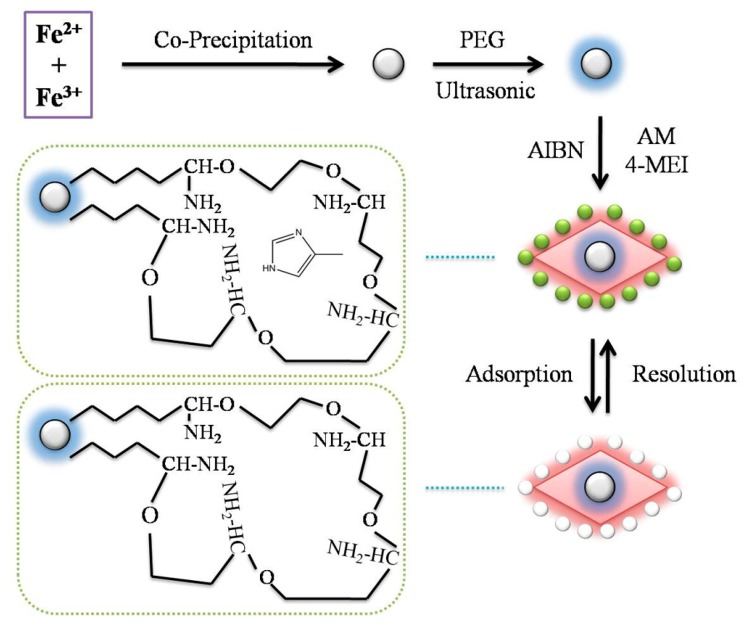
Schematic illustration for the synthetic route of 4-MEI-MMIPs.

Figure 1 showed the schematic procedure of preparing Fe_3_O_4_ MMIPs*.* The particles were synthesized by the coprecipitation method. Due to their low compatibility with the organic phase, the surface of Fe_3_O_4_ particles was modified to endow with hydrophobicity for further encapsulation. The polymeric chains were introduced onto the surface of the Fe_3_O_4_ particles by PEG as a surface modifier. As a result, the Fe_3_O_4_ particles were encapsulated in suspended droplets [23]. The molecular recognition of the template molecules is based on the intermolecular interactions between template molecules and functional groups [24]. Monomers play an important role in the generation of recognition sites through self-assembled with the template.

Monomer selection test was carried out between activated monomers (AM) and methacrylic acid with same amount. As a result, AM showed better molecular recognition ability in polar condition. According to the literature [22], MMIPs were synthesized at the optimum molar ratio of 1:6:30 [template:AM:EGDMA]. In order to improve the stability of the synthesized polymers, St was chosen as a copolymer monomer to introduce unsaturated bonds and achieve the formation of cross-linked backbone chain within the polymer network. Then, the polymer could be prepared reproducibly with good homogeneity and density.

**Figure 2:**
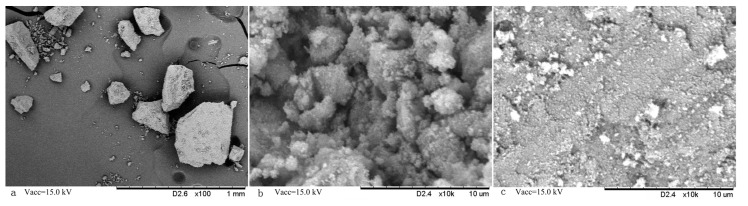
Scanning electron micrographs of the MMIPs and MNIPs: (**a**) MMIPs; (**b**) surface of MMIPs; (**c**) surface of MNIPs.

Figure 2 showed the surface morphologies of MMIPs and MNIPs by SEM. Fe_3_O_4_ beads were wrapped in irregular particles. The dimension of the particles ranged from 100 µm to 400 µm (Figure 2a). The surface of the MMIP (Figure 2b) showed rougher and more porous than that of MNIPs (Figure 2c). Some speckles and cavities were observed within the MMIP because of the addition and elution of template molecules. It indicates that imprintings were formed within the prepared MMIP. This specific structure is conducive to the adsorption or desorption of the template molecules from MMIPs.

**Figure 3:**
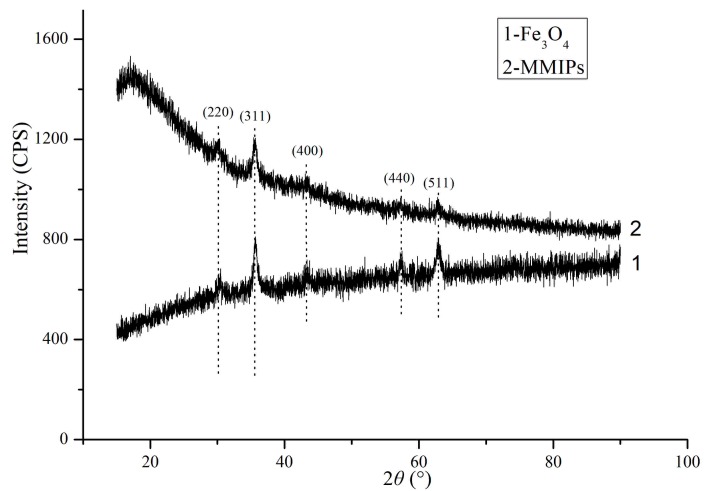
XRD patterns for the pure Fe_3_O_4_ (1) and MMIPs (2).

XRD test was carried out to analyze the components of the magnetic nanoparticles and MMIPs (Figure 3). According to the crystal planes with cubic crystal structures, it can be inferred that the magnetic nanoparticles and polymers were composed of Fe_3_O_4_. The crystallographic structures of Fe_3_O_4_ nanoparticles and magnetic imprint polymers remain essentially.

**Figure 4:**
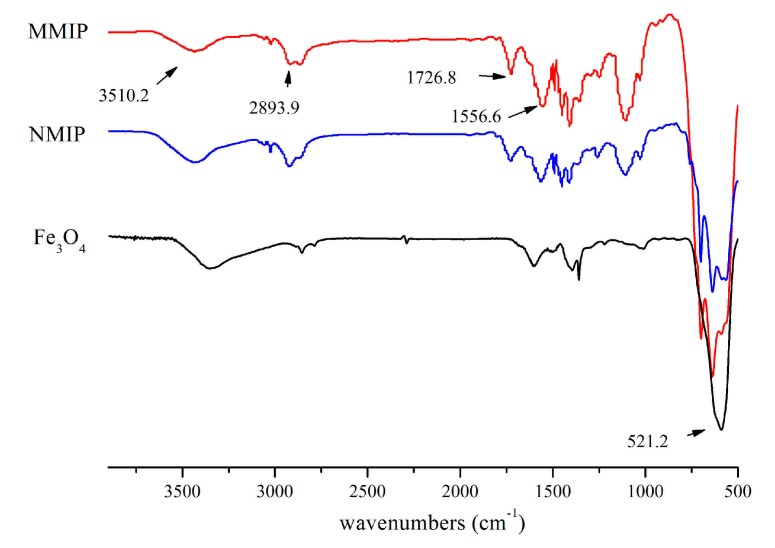
FT-IR spectra of the MMIP, NMIP and Fe_3_O_4_.

In order to further ensure the synthesis process, FT-IR was conducted for Fe_3_O_4_, MMIPs, and MNIPs. In comparison with Fe_3_O_4_ nanoparticles, the absorption band at 521.2 cm^−1^ was attributed to Fe-O bond; it revealed that Fe_3_O_4_ was embedded in these polymers (Figure 4). The characteristic band for the C-H aromatic stretching vibrations of styrene units was found at 2893.9 cm^−1^. The typical peak at 3510.2 cm^−1^ was caused by the N-H stretching of AM. Because most AM were cross-linked, the absorbance peak of C=C at 1556.6 cm^−1^ was weakened. In the MMIPs spectrum, the absorbance peak at 1726.8 cm^−1^ attributed to C=O revealed that the MMIPs were polymerized by EGDMA and AM. Additionally, by reason of the hydrogen-bond interaction between 4-MEI and AM, the peak strength of MNIPs was lower than that of MMIPs.

In the following, VSM was carried out to analyze the magnetic properties of Fe_3_O_4_ nanoparticles and MMIPs. The saturation magnetizations were 67.34 and 3.91 emu g^−1^ for Fe_3_O_4_ nanoparticles and MMIPs, respectively. The Ms value decreased due to the comparatively low content of Fe_3_O_4_ nanoparticles loaded on MIPs. On one hand, the results suggested that the typical superparamagnetic behaviors of MMIPs sorbents and Fe_3_O_4_ could avoid the aggregation. On the other hand, MMIPs displayed relatively high saturation magnetizations. As a result, it is easy to separate the MMIPs from the sample solution by an external magnet.

### 3.2. Binding Isotherms 

**Figure 5:**
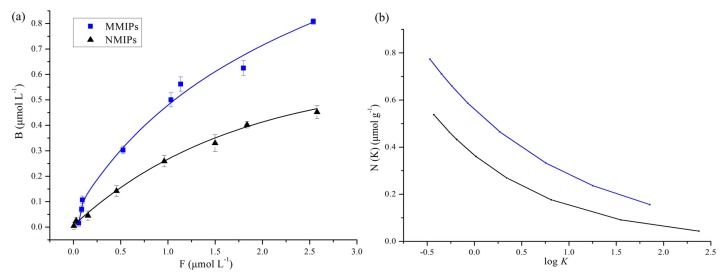
(**a**) 4-MEI adsorption isotherms for MMIPs and MNIPs with the corresponding experimental FI for MMIPs and MNIPs; (**b**) affinity distributions of MMIPs (thick line) and MNIPs (thin line).

Figure 5a summarized the isothermal adsorptions and experimental FI of 4-MEI in the MMIPs and MNIPs. Although the binding amount of 4-MEI increased with the increase of the initial concentration on both synthesized sorbents, MMIPs exhibited higher affinity than MNIPs.

In order to analyze the binding data, the FI affinity distribution analysis model was involved.

For heterogeneous surfaces, the FI model is regarded as the most important multilayer adsorption isotherm [25].

(1) logB=mlogF+loga

where *B* is the concentrations of bound analytes, *F* is the concentrations of free analytes, and *m* is the heterogeneity index. Ranging from 0 to 1, the parameter *m* increases along with the decreasing heterogeneity of the material. Value 1 indicates homogeneous and value 0 indicates increasingly heterogeneous. The lower value of *m* is, the higher percentage of high-affinity binding sites has. As a result, MMIPs represented higher affinity than MNIPs according the *m* value in Table 1. 

According to Equation (2) and the parameters (*a* and *m*) from Equation (1), the affinity distribution can be calculated [25].

(2) $$N(k)=2.303am(1-m^{2})K^{-m}$$

where *K* is the affinity constant (*K* can be assumed equal to 1/*F*), and *N*(*k*) is the number of binding sites with a given affinity.

*N_k_*_min-*k*max_ represents the number of binding sites in material per gram (see Equation (3)), and *K_k_*_min-*k*max_ represents the weighted average affinity constant (see Equation (4)). Both of them can be calculated [26,27]:

(3) $$N_{k\min-k\max}=a(1-m^{2})(K_{\min}^{-m}-K_{\max}^{-m})$$

(4) $$K_{k\min-k\max}=\left(\frac{m}{m-1}\right)\left(\frac{K_{\min}^{1-m}-K_{\max}^{1-m}}{K_{\min}^{-m}-K_{\max}^{-m}}\right)$$

*K*_min_ and *K*_max_ can be considered as the corresponding reciprocal concentrations *K_min_* = 1/*F_max_* and *K_max_* = 1/*F_min_*. Within the limits of the two variables, the values for these parameters in Equations (3) and (4) can be calculated from the experimental maximum (*F_max_*) and minimum (*F_min_*) free analyte concentrations.

The distribution of MMIPs and MNIPs binding affinities was shown in Figure 5b in the form of a common semi logarithmic format (*N* vs. log *K*). The site-energy distribution of the polymers could be inferred from this plot. Compared with MNIPs, the number of binding sites in the MMIPs was more, regardless of tested concentrations. This result proved the imprinting behavior.

Table 1 summarized the calculated fitting parameters. For MMIPs and MNIPs, the weighted average *K_kmin-kmax_* were calculated to be 13.02 and 2.56 L mmol^−1^, respectively, while the total average *N_kmin-kmax_* were 3.55 and 0.89 μmol g^−1^, respectively. The affinity constants and the total number of binding sites in MMIPs were more than that in MNIPs. These results suggested that the template molecules play a significant role in the formation of specific recognition sites during the imprinting behavior.

### 3.3. Selectivity Evaluation of MMIPs and MNIPs

**Figure 6:**
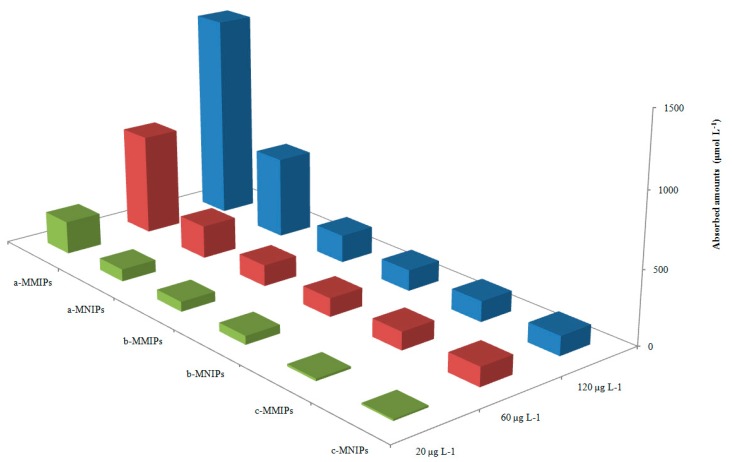
Selective recognition capability of MMIPs and MNIPs to (a) 4-MEI, (b) salicylic acid, and (c) benzoic acid at concentrations of 20, 60 and 120 mg L^−1^.

Compared with two reference molecules, Figure 6 illustrates the adsorption ability of MMIPs and MNIPs, based on three concentration levels of the standard solution (20, 60, and 120 μg L^−1^). The binding ability of the MMIPs to 4-MEI was significantly stronger than that of salicylic acid and benzoic acid. Three parameters were used to evaluate the selectivity of MMIPs: the selectivity coefficient (*k*), distribution coefficient (*K_d_*), and relative selectivity coefficient (*k’*). The selectivity coefficient (*k*) is defined as the ratio of the target *K_d_* to the competitive molecule [28]. The distribution coefficient (*K_d_*) is defined as the concentration ratio of adsorbed-to-unadsorbed analyte. *K_d_* value indicates the adsorption ability of polymer materials. The more *K_d_* value is, the greater the adsorption capacity is. 

The relative selectivity coefficient (*k’*) is defined as the ratio of the *k* values of target-to-competitive molecule. *K_d_* denotes the adsorption capacity [29]. The larger the value of *K_d_* indicates the substance possesses stronger adsorbability. The parameter*k* reveals the selectivity between two substances. The parameter *k’* indicates the selective diversity between MMIPs and MNIPs. The larger value of *k’* reflects the greater selectivity of molecular imprinting.

Table 2 showed the three parameters for the tested compounds. There were no obvious differences among the three compounds for MNIPs. However, the MMIPs exhibits significant adsorption selectivity for 4-MEI. This result reveals that the prepared MMIPs possesses higher selectivity for 4-MEI, compared to the reference compounds. 

### 3.4. Optimization of Extraction and Desorption Time 

**Figure 7:**
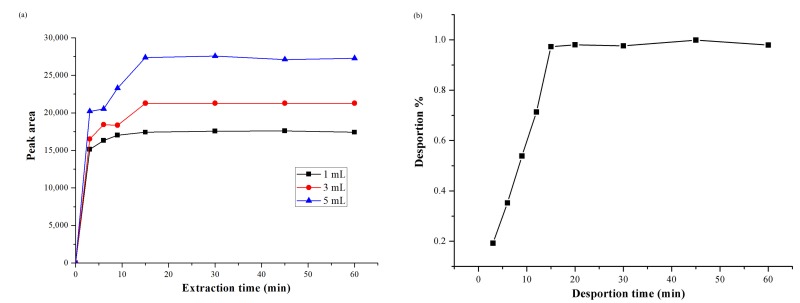
(**a**) Dynamic adsorption isotherms of MMIPs of 4-MEI in three sample volumes (i.e., 1, 3, and 5 mL); (**b**) effect of desorption time.

Three different volumes (1, 3, and 5 mL) were set to investigate the factors which could affect the extraction process. The concentration of 4-MEI was 300 ng mL^−1^. Figure 7a showed that the time of adsorption equilibrium was about 15 min. The sample volume played an important role in the bonding amount of targets. As a result, the adsorption volume and extraction time were set to 1 mL and 15 min, respectively. 

In order to investigate the desorption time of the target analytes, different time intervals (3, 6, 9, 15, 30, and 60 min) were set. Figure 7b revealed that the analyte can be completely desorbed in 15 min. After desorption, a magnet could separate the MMIPs from the solution in a short time (about 30 s). Hence, the desorption time was set at 15 min.

### 3.5. Validation of the Analytical Method 

A series of experimental parameters were evaluate to validate the analytical methodology, including linear range, correlation coefficient, limit of detection (LOD), and limit of quantification (LOQ). The linear regression method was used to make the standard curve, by plotted peak areas versus concentrations. The regression equation was *y* = 635.6 + 0.92*x* (*r* = 0.9977), and the concentration ranged from 5.7 to 1148 µg L^−1^. LOD and LOQ were 1.71 and 5.64 µg L^−1^, by defined as 3 and 10 times the signal to noise ratio, respectively.

In order to evaluate the repeatability, accuracy and recovery of the method, standard addition method was used. With three concentration levels, 4-MEI was spiked into original sample solutions. Table 3 showed that the recovery rates of the spiked samples ranged from 97.33% to 104.57% within RSD range of 0.158% to 2.38%. These results revealed that this analytical method was sensitive and reliable.

### 3.6. Analysis in Real Samples

**Figure 8:**
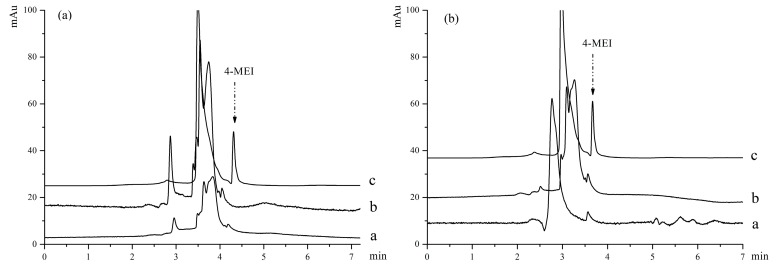
Chromatograms of two samples: (**a**) represents sample Shengchou; (**b**) represents sample Laochou: a—initial solutions; b—solution extracted with MNIPs; c—solution extracted with MMIPs.

The study intended to provide a simple, practical, and selective procedure which can be applied to detect the analytes in complex samples by MMIPs. According to the procedure mentioned in Section 2.5, two soy sauce samples were extracted with MMIPs and MNIPs. The chromatogram of analysis results were showed in Figure 8, including direct injections of the two soy sauce samples, samples extracted by MMIPs and MNIPs. The results indicated that 4-MEI cannot be detected directly from the soy sauce samples by HPLC without enrichment (Line a). No related peak was obtained (Line b) in the MNIPs extracted samples. This result indicated that 4-MEI could not be extracted from soy sauce samples by MNIPs. 4-MEI in the soy sauce samples was determined successfully and selectively by the prepared MMIP extraction method (Line c). But unfortunately, by this efficient method, contents of 4-MEI were found to be 0.058 and 0.108% in the real soy sauce samples. This result is consistent with facts that the color of Shengchou is lighter than Laochou. More caramel is put into Laochou because the main function of Laochou soy sauce is to color the food in Chinese cuisine. The contents of 4-MEI in the samples exceeds the safe limit according to China’s national standard. As a result, further health risk assessment should be conducted for the unqualified soy sauce.

## 4. Conclusions

In order to selectively extract 4-MEI in soy sauce samples, MMIPs were prepared for the HPLC analysis. The proposed MMIPs were characterized by SEM, VSM, FT-IR, and XRD. This magnetic polymer showed satisfied selectivity recognition properties and high adsorption capacity to the target template molecule. During a short extraction procedure, adsorption and desorption equilibrium were reached, and MMIPs could be collected quickly by a magnet after extraction. This method could be applied to the determination of 4-MEI in soy sauce samples which attribute to the good selectivity and specificity of MMIPs. The validity of this analysis method was proved by the high recovery rates. There is a perspective potential for MMIPs in determining 4-MEI in soy sauce samples.

## Figures and Tables

**Table 1:** Freundlich fitting parameters, number of binding sites (*N*_kmin-kmax_) and weighted average affinity (*K_k_*_min-*k*max_) for 4-methylimidazole MMIPs and MNIPs.

Fitting Parameters	MMIPs	MNIPs
*N_k_*_min-*k*max_ (µmol g^−1^)	3.55	0.89
*K_k_*_min-*k*max_ (L mmol^−1^)	13.02	2.56
α	0.48	0.89
m	0.39	0.83

**Table 2:** Recognition properties of MMIPs and MNIPs ^a^.

	Levels (μg L^−1^)	*K_d_* (μmol g^−1^)			*k*		*k*’	
		*K_d_*_1_ 4-MeI	*K_d_*_2_ Salicylic Acid	*K_d_*_3_ Benzoic Acid	*k*_1_	*k*_2_	*k*’_1_	*k*’_2_
MMIPs	120	23.20	3.98	4.99	5.83	4.65	7.29	7.01
	60	15.01	4.17	3.09	3.60	4.86	2.19	4.72
	20	10.11	1.56	2.52	6.48	4.01	8.85	6.15
NMIPs	120	3.74	4.68	5.64	0.80	0.66		
	60	3.79	2.31	3.68	1.64	1.03		
	20	1.26	1.72	1.93	0.73	0.65		

^a^ *K****_d_***, distribution coefficient; *k*, selectivity coefficient; *k*_1_ = *K_d_*_1_/*K_d_*_2_, *k*_2_ = *K_d_*_1_/*K_d_*_3_; *k*’, relative selectivity coefficient; *k’*_1_ = *k*_1MMIP_/*k*_1MNIP_, *k’*_2_ = *k*_2MMIP_/*k*_2MNIP_.

**Table 3:** Accuracy of the method for sample solutions spiked at different concentrates (*n* = 3).

Analyte	Added (µmol L^−1^)	Found (µmol L^−1^)	Recovery (% ^a^)	Average (%)	RSD (%)
4-MEI	109.8	111.2	101.29	101.11	0.158
	109.8	110.9	101.07		
	109.8	110.8	100.98		
	4.3	4.5	104.57	102.29	2.387
	4.3	4.4	102.57		
	4.3	4.3	99.71		
	0.9	0.89	98.67	97.94	0.784
	0.9	0.88	97.82		
	0.9	0.87	97.33		

^a^ [(Found-base)/added] × 100%.
